# *Origanum dictamnus* Oil Vapour Suppresses the Development of Grey Mould in Eggplant Fruit* In Vitro*

**DOI:** 10.1155/2014/562679

**Published:** 2014-09-01

**Authors:** Andriana Stavropoulou, Kostas Loulakakis, Naresh Magan, Nikos Tzortzakis

**Affiliations:** ^1^Department of Agriculture, School of Agriculture Food and Nutrition, Technological Education Institute of Crete, Stavromenos, 71004 Heraklion, Greece; ^2^School of Applied Sciences, Cranfield University, Bedfordshire MK43 0AL, UK; ^3^Department of Agricultural Sciences, Biotechnology & Food Science, Cyprus University of Technology, 3036, Limassol, Cyprus

## Abstract

Grey mould rot (*Botrytis cinerea*) development *in vitro* or in eggplant (*Solanum melongena* L.) fruit was evaluated after treatment with dittany (*Origanum dictamnus* L.) oil (DIT) and storage at 12°C and 95% relative humidity during or following exposure to the volatiles. DIT volatiles used in different concentration (0-50-100-250 *μ*L/L) and times of exposure (up to 120 h) examined the effects on pathogen development as well as fruit quality parameters. *In vitro,* fungal colony growth was inhibited with the application of DIT oil (during or after exposure) and/or time of application. Continuous exposure to oils reduced conidial germination and production with fungistatic effects observed in 250 *μ*L/L. *In vivo,* fungal lesion growth and conidial production reduced in DIT-treated fruits. Interesting, in fruits preexposed to volatiles before fungal inoculation, DIT application induced fruit resistance against the pathogen, by reduced lesion growth and conidial production. Conidial viability reduced in >100 *μ*L/L DIT oil. Fruits exposed to essential oil did not affect fruit quality related attributes in general, while skin lightness (*L* value) increased in 50 and 100 *μ*L/L DIT oil. The results of the current study indicated that dittany volatiles may be considered as an alternative food preservative, eliminating disease spread in the storage/transit atmospheres.

## 1. Introduction

The economical impacts of spoiled foods and the consumer's concerns over the safety of foods containing synthetic chemicals, as well as the rise in the consumption of fresh produce over the last decades, have driven demand for improved commercial storage/transit conditions, in order to control postharvest disease proliferation and maintain fruit quality. Indeed it is a common practice the use of chemical treatments to preserve fresh produce, including chlorine- (or bromine-) based disinfectants. However, growing health and environmental concerns have arisen over current sanitation practices because of potentially carcinogenic residues [1] resulting from agrochemical inputs, as well as their ineffectiveness on a wide range of microorganisms [2].

A lot of attention has been paid to naturally derived compounds or natural products [3]. Most of the natural products are readily available, environmentally safe, with low risk for developing resistance to pests, less hazardous to nontarget organisms and pest resurgence, with less adverse effect on plant growth, and, above all, occasionally less expensive than some of the chemical treatments [4]. Recently, there has been considerable interest in extracts and essential oils (EO) from aromatic plants with antimicrobial activities for controlling pathogens and/or toxin producing microorganisms in foods which are considered as human-safe and environmentally friendly [3, 5–7] and can be ideal candidates for use as agrochemicals [8–10].

The advantage of essential oils is their bioactivity in the vapour phase, a characteristic that makes them useful as possible fumigants for stored commodity protection [11]. Several studies reported the effectiveness of essential oil on fresh produce preservation such as sweet cherry (*Prunus avium *L.), banana (*Musa *spp.), kiwifruit (*Actinidia chinensis* L.), grape (*Vitis vinifera* L.), pear (*Pyrus communis* L.), avocado (*Persea americana *L.), strawberry (*Fragaria vesca* L.), and tomato (*Solanum lycopersicum* L.) fruit [11–17]. Such new treatments could have relatively high acceptance because of consumer's preference for natural plant products, for example, essential oils compared with synthetic fungicides [15]. However, evaluation of volatile concentrations is needed which may improve the suppression of fungal development* in vivo*, initiating defence-related mechanism of the fruit and avoiding problems associated with reactive oxygen species (ROS) accumulation.

Eggplant is wide consumed vegetable worldwide and has been associated with cell membrane protection as containing “nasunin” a potent antioxidant and free radical scavenger as well as numerous vitamins and minerals, such as B1, B6, folate, copper, manganese, and potassium [18]. In animal studies, “nasunin” has been found to protect the lipids (fats) in brain cell membranes. Indeed, many aspects encompass fruit quality and include not only firmness, flavour, colour, and nutritional value, but also shelf life, processing attributes, and resistance to pathogens [19]. The effect of natural volatiles on fresh produce preservation was investigated on only a few commodities (i.e., papaya [20], pear [14], kiwifruit [21], tomato, and strawberry [16, 17]).

The susceptibility of freshly harvested produce to microbial infection increases during storage as a result of physiological changes that facilitate pathogen development [22]. Several pre- and postharvest technologies have been used to control decay, with pathogenic fungi alone causing nearly 20% reduction in the yield of major food and cash crops. Grey mould rot [*Botrytis cinerea* Pers.: Fr (teleomorph:* Botryotinia fuckeliana*)] is a common and wide spread rot of ripe and/or overripe fresh produce [23].

This study was undertaken to determine for the first time if essential oil of dittany would reduce spoilage by* B. cinerea* in eggplant fruit as well as the effects of fruit quality parameters.

## 2. Materials and Methods

### 2.1. Plant Material and Inocula

Eggplant (*Solanum melongena* L., cv. Vernina) fruit obtained directly from the field (organic cultivated in a clay loam soil, frequently irrigated according to crop needs), Heraklion, Greece. Fruit, following harvest, were randomized in uniform size, color, and ripeness (dark purple stage) and were free from defect or injury and then were used for experimental needs as described in Table 1.

Organic essential oilsderived from dittany (*Origanum dictamnus *L.), obtained from a crop in Heraklion prefecture (harvested during the early summer period while plants were in full bloom) were extracted by hydrodistillation (Clevenger apparatus for 3 h). The analysis of the essential oil was performed using a Shimadzu (QP 5050A) GC, equipped with a SBP-5 capillary column (30 m, 0.25 mm i.d., 0.25 mm film thickness) and a quadrupole mass spectrometer as detector. The carrier gas was helium, at a rate of 0.9 mL/min. Column temperature was initially maintained for 5 min at 50°C, then gradually increased to 150°C at a rate of 5°C/min and kept for 10 min, and finally increased to 280°C at 5°C/min and held for 20 min. For GC-MS detection an electron ionization system was used with ionization energy of 70 eV. The sample was measured in a split mode procedure with a split ratio 1 : 42. 5 *μ*L of oil sample was dissolved in 1 mL diethyl-ether and 1 *μ*L was used as an injection volume. Injector and detector (MS transfer line) temperatures were set at 230°C and 250°C, respectively. The scanning range was 30–700 m/z. The quantification of the components was based on the total number of fragments (total ion count) of the metabolites, as detected by the mass spectrometer. The identification of the chemical components was carried out based on the retention time of each component (Rt) compared with those of commercially available compounds, by analysis of their mass spectra, by the use of the NIST21, NIST107, and PMWTOX2 mass spectra libraries, and by comparison with the literature data [24]. Calculation of retention indices was performed in accordance with the work of van den Dool and Dec. Kratz [25], in comparison to the retention times of standard hydrocarbons (C9–C25). Also, when necessary, coinjection with standard compounds was carried out. The composition of the essential oil is presented in Table 2.

*Botrytis cinerea (BPIC 2585) *cultures were obtained from the Benaki Phytopathological Institute in Athens, Greece (http://en.bpi.gr/). Isolates were aseptically subcultured and purified by serial transfers onto standard triple-vented Petri dishes containing 20 mL of potato dextrose agar (PDA, Oxoid Ltd., Hampshire, England). Plates were incubated in the dark at 25°C for 1 week and cultures were stored at 4°C for long-term use. Fresh cultures were prepared for each experiment and were left to grow in the dark for 5–7 days at 25°C in an incubation chamber, before being used in each of the* in vitro *experiments.

### 2.2. Treatments with Volatile Compounds

Dittany (DIT) volatiles (0-50-100-250 *μ*L/L) used in this study were diluted in distilled H_2_O with 5% (v/v) Tween-20. Aliquots (0.1 mL) of each volatile solution were placed into individual filter paper (3.5 × 3.5 cm) placed on the inverted lid of each Petri dish; the lids were closed and sealed with parafilm. Fruits were placed into 5.4 L polystyrene containers with snap-on lids for each individual experiment. Aliquots (5 mL) of each volatile solution were placed into individual filter paper (3.5 × 3.5 cm) placed on the inverted lid of each Petri dish which were subsequently placed inside the plastic containers. Wet filter paper was placed in each container to maintain high relative humidity (RH ~ 95%) during the storage period. The experimental design of DIT treatments is presented in Table 1. The volatiles were allowed to vaporize inside the containers spontaneously at 20°C for 2 h. The containers were then stored at 12°C. Control samples were handled similarly with the exception of the volatile treatment [controls consisted of distilled H_2_O with 5% (v/v) Tween-20]. The experiments were repeated twice.

### 2.3. Impacts of Volatile Enrichment on Pathogen Development* In Vitro*

A mycelial plug (5 mm diameter) obtained from the periphery of 4-5-day-old culture of* B. cinerea *at 25°C was placed in the centre of plates containing PDA. Following inoculation plates were incubated for 5 d exposed to ambient air (AA) or DIT volatiles for different times (24, 48, or 120 h) in the dark at 12°C/95% RH (sustained effect). In a second experiment, examining the putative fungistatic or fungicidal effects of DIT vapours on* B. cinerea*, the original inoculum was taken from each plate and transferred to fresh PDA medium, while plates were incubated for 5 d. In a third experiment, plates containing PDA medium were exposed to AA or volatiles for 24, 48, or 120 h, prior to inoculation. Following exposure, culture medium was inoculated (as described above), lids were replaced on the plates, and plates were transferred to AA for additional 5 d (“memory effect”). Colony development (colony diameter) was monitored and data was expressed as colony area (cm^2^).

### 2.4. Impacts of Volatile Enrichment on Grey Mould Development in Wound-Inoculated Fruit

Mature eggplants fruits were selected for uniformity in size, appearance, and the absence of physical defects. One wound (4 mm diameter and 2-3 mm deep) was made using a sterilised spike. A mycelial plug (3.5 mm diameter) was removed from the advancing margins of a 4-5-day-old culture of* B. cinerea* and it was placed inside of the superficial wound of the fruit. The following experiments took place as described in Table 1.

- Eggplant fruits inoculated with* B. cinerea* were placed in containers and exposed to AA or volatiles for up to 14 d (sustained effect).
- Eggplant fruits first were exposed on AA or volatiles for 5 d, then inoculated with* B. cinerea,* and transferred to AA for an additional 7 d (“memory effect”).

Fumigation was performed in the dark at 12°C, 95% RH. Lesion area growth (cm^2^) was monitored at 7 d and/or 14 d.

### 2.5. Effect of Enrichment with Volatiles on Spore Production

Plates (PDA medium) were inoculated centrally with a mycelial plug (5 mm diameter) of* B. cinerea, *placed in containers, and exposed to AA or DIT volatiles for 14 d (sustained effect). In a second experiment, plates were placed in containers, exposed to AA or volatiles for 5 d, and then inoculated with the fungal mycelium plug. Following exposure, lids were replaced and the plates were transferred to AA for 14 d (memory effect). Following exposure to volatiles or AA, spores were collected with an L-shaped spreader with 15 mL dH_2_O (with Tween 80; 0.1% v/v) for 5 min. The spore suspension was concentrated to a final 1 mL volume and a haemocytometer slide was used to count the spores.

Eggplant fruits were wounded and inoculated with mycelium plug of* B. cinerea *as described previously. Fruits were placed in the containers and exposed to AA or volatiles for 14 d until spores formed. In a second experiment, fruits were exposed to AA or volatiles for 5 d, inoculated with* B. cinerea *(as above), and transferred to AA for 14 d until spores formed. Fumigation was performed in the dark at 12°C, 95% RH. Following exposure to volatiles or AA, spores were collected from inoculated fruits by washing the decayed surface of fruits with 10 mL (0.1% v/v) Tween 80 using a sterile L-spreader to rub the surface (3 times). The solutions were filtered using sterile gauze to remove any conidiophores or mycelium. Spore suspension was concentrated to final 1 mL volume. A haemocytometer slide was used for a direct microscope counts to quantify spore production.

### 2.6. Effect of Enrichment by Volatiles on Spore Germination

Spores germination studies were conducted in several treatments as follows.

- Spores from 14-day-old* B. cinerea *colonies exposed to AA or volatiles as described previously (2.3) were collected with an L-shaped spreader and inoculated (300 *μ*L) onto thin layer PDA medium (2-3 mm thick). Inoculated Petri dishes (6 replicates) were incubated at 12°C for 12–24 h until the conidia in the controls had germinated forming distinguished germ tubes (approximately twice in size as the size of conidia). Experiments were then terminated and germination was stopped by adding 100 *μ*L of formaldehyde on filter papers fixed to the inside of Petri lids. Plates were sealed, inverted, and left at ambient temperature overnight, and then germination could be scored at will.
- Spores from 14-day-old* B. cinerea *colonies preexposed to AA or volatiles as described previously (memory effect) were collected with an L-shaped spreader and inoculated onto PDA medium (2-3 mm thick). Plates were incubated at 12°C for 12–24 h until the conidia in the controls had germinated forming distinguished germ tubes.
- Spore suspension was extracted from inoculated and volatile-exposed eggplant fruits (see Section 2.4 (a), (b)), inoculated on thin layer PDA medium, and incubated to AA for 12–24 h in the dark at 12°C/95% RH. Measurements were recorded after 24 h incubation. Within each replicate (12), 100 spores were examined and the percentage of germinated spores was calculated.

### 2.7. Effect of Volatiles Enrichment on Fruit Quality Related Parameters

Healthy (not infected) fruits were labeled and the weight was recorded prior to exposure to AA or DIT volatiles. Fruits were weighed after 7 d and percent weight loss of original weight was computed. Fruit firmness was measured at 2 points on the shoulder of eggplant fruit (skin removed), respectively, for each treatment by applying a plunger of 8 mm in diameter, using a Chatillon DPP Dial Push-Pull Gauze (Chatillon, France). The amount of force (Kg cm^−2^) required to break the radial pericarp (i.e., surface) of each eggplant was recorded at ambient (22–24°C) temperature. Fruit color measurements (*L*, *C*, *h* values) were taken around the fruit equator (2 measurements per fruit) for calyx, skin, and pulp with a Minolta Chroma Meter CR400 (Konica Minolta, Japan). Total soluble solids (TSS) concentration was determined on the fruit juice for each treatment with a digital refractometer PR-1 (ATAGO, Japan) at 20°C and results were expressed as the mean (%) of °Brix. Subsamples of homogenized fruit tissue were used to determine the pH of fruit juice using a standard pH meter (InoLAB, pH level 2, WTW, Germany). Titratable acidity (TA) was determined by potentiometric titration, using fruit samples (5 mL) diluted in 20 mL distilled water and titrated with 0.1 N NaOH and monitored up to 8.2 end point with a pH meter. The reported values were expressed in terms of citric acid percentage. Fruit respiration rates were recorded daily with Handheld Gas Analyser, Check Point O_2_/CO_2_ PBI (Dansensor, Denmark).

### 2.8. Statistical Analysis

Data were first tested for normality and then subjected to analysis of variance (ANOVA). Sources of variation were time of storage and treatments. Significant differences between mean values were determined using Duncan's multiple range test (*P* = 0.05) following one-way ANOVA. Significant differences on percentage values (spore germination) were logarithmic transformed prior using ANOVA. Statistical analyses were performed using SPSS (SPSS Inc., Chicago, USA) and graphs were produced using Prism v.2.0 (Graph Pad Inc., San Diego, USA).

## 3. Results and Discussion

### 3.1. Impact of Volatile Vapour Enrichment on Gray Mould Development* In Vitro*

**Figure 1:**
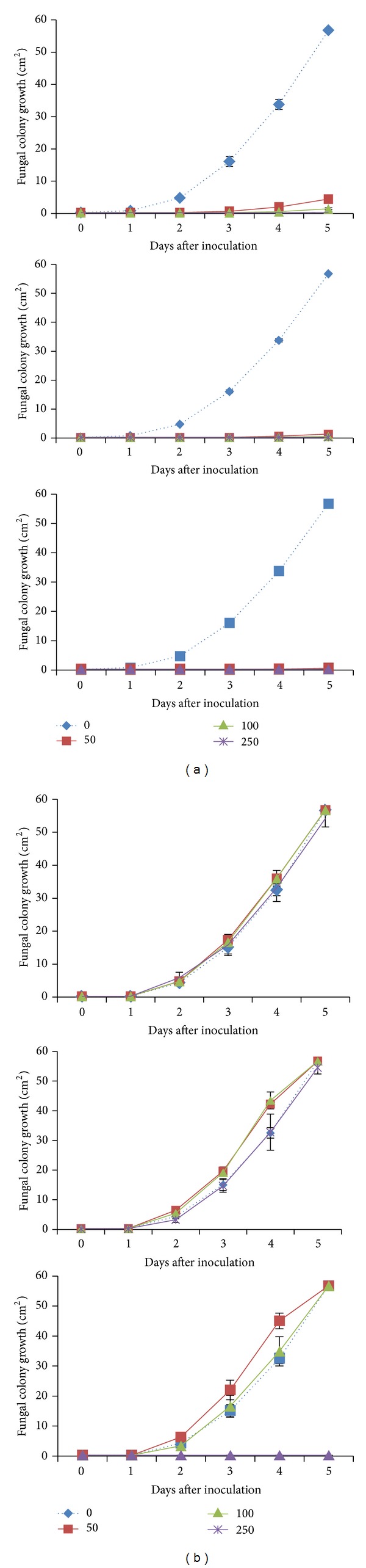
Impacts of dittany essential oil-enrichment [(control: - -**◆**- -) or volatiles; 50 *μ*L/L (**—■—**), 100 *μ*L/L (**—▲—**), and 250 *μ*L/L (**—**∗**—**)] on colony development (cm^2^) of grey mould (*Botrytis cinerea*) raised and exposed to dittany vapors on PDA (a) during volatiles exposure or (b) following transfer to fresh PDA medium. Plates were maintained throughout at 12°C and 95% RH. Values represent mean (±SE) of measurements made on six (a) and three (b) independent plates per treatment.

**Figure 2:**
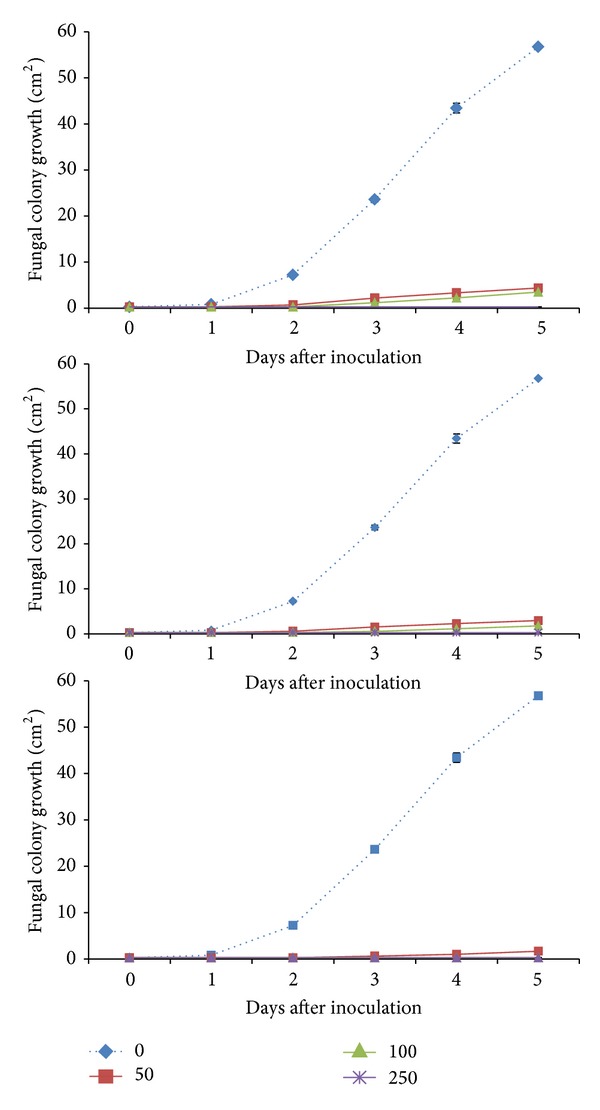
Impacts of dittany essential oil-enrichment [(control: - -**◆**- -) or volatiles; 50 *μ*L/L (**—■—**), 100 *μ*L/L (**—▲—**), and 250 *μ*L/L (**—**∗**—**)] oncolony development (cm^2^) of grey mould (*Botrytis cinerea*) on pre-exposed PDA to dittany vapors and then inoculated with fungus. Plates were maintained throughout at 12°C and 95% RH. Values represent mean (±SE) of measurements made on six independent plates per treatment.

Culture PDA media with dittany oil-enrichment resulted in significant (*P* < 0.05) reduction on subsequent colony (vegetative phase) development of* B. cinerea *(between 92 and 99%) and this was evidence even for short (24 h) exposure with DIT (Figure 1). When inocula were transferred to fresh PDA media, fungal colony developed equally well in DIT-treated and nontreated media, implicating the fungistatic effects of DIT. Similarly, in preexposed PDA to DIT volatiles, vapour enrichment suppressed fungal colony growth after vapour exposure (Figure 2), independently of concentration and duration of DIT exposure. Soliman and Badeaa [5] found that ≤500 *μ*L/L of cinnamon (*Cinnamomum zeylanicum* L.) oil can inhibit* Aspergillus flavus*,* Aspergillus parasiticus*,* Aspergillus ochraceus,* and* Fusarium moniliforme* on PDA, being in accordance with the present findings.* In vitro* studies of oregano (*Origanum vulgare* L.), thyme (*Thymus capitatus* L.), lemongrass (*Cymbopogon citratus* L.), and cilantro (*Coriandrum sativum* L.), vapours (500–1000 *μ*L/L) showed complete growth inhibition of* B. cinerea *and* Alternaria arborescens* [26].

The impact of DIT oil enrichment on fungal sporulation and spore germination (reproductive phase)* in vitro* revealed both spore germination and production to be significantly (*P* < 0.05) inhibited when compared with equivalent plates stored in ambient air (Table 3). Spore production was depressed by 100% for* B. cinerea*, in sustained vapour-treated spores or vapour preexposed PDA medium for >100 *μ*L/L of DIT application while significant effects were observed in low (i.e., 50 *μ*L/L) DIT concentration in vapour preexposed PDA medium. Spore germination failed to be measured in >100 *μ*L/L of DIT application due to spore production limitations. Previous studies reported that the sporulation of* Colletotrichum coccodes*,* B. cinerea*,* Cladosporium herbarum, Rhizopus stolonifer,* and* Aspergillus niger* was inhibited by lemongrass or cinnamon oil [9, 27] while* A. flavus* was completely inhibited by* C. citrates *(2800 *μ*L/L) when used as fumigant whereas aflatoxin production was inhibited at 100 *μ*L/L of* C. citrates* treatments [28].

### 3.2. Effects of Volatile Vapour Enrichment on Gray Mould Development* In Vivo*

**Figure 3:**
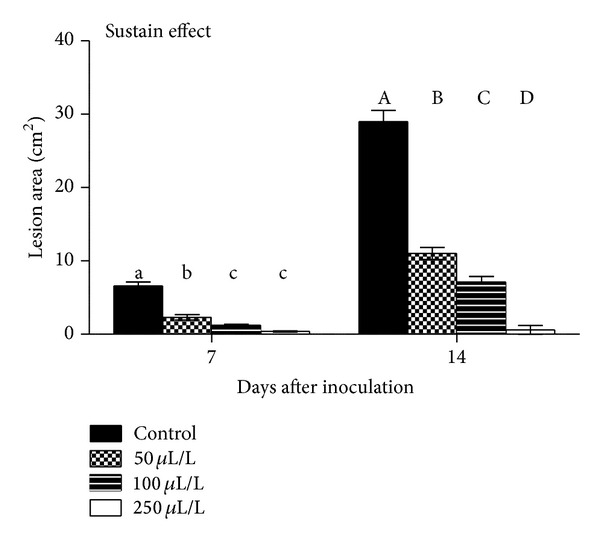
Impacts of dittany essential oil-enrichment (control or volatiles; 50-100-250 *μ*L/L) on eggplant lesion area (cm^2^) of grey mould (*Botrytis cinerea*). Fruits were maintained throughout at 12°C and 95% RH. Values represent mean (±SE) of measurements made on twelve independent fruits per treatment. Treatments followed by the same letter do not differ significantly at *P* = 0.05 according to Duncan's range test.

**Figure 4:**
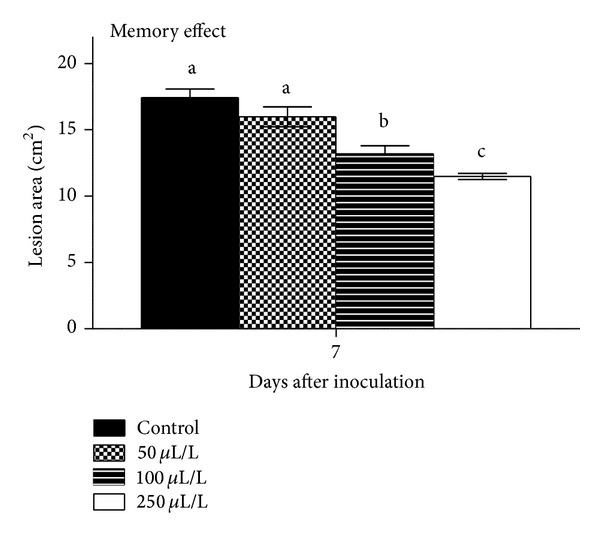
Impacts of dittany essential oil-enrichment (control or volatiles; 50-100-250 *μ*L/L) on preexposed eggplant fruits to volatiles, on lesion area (cm^2^) of grey mould (*Botrytis cinerea*). Fruits were maintained throughout at 12°C and 95% RH. Values represent mean (±SE) of measurements made on twelve independent fruits per treatment. Treatments followed by the same letter do not differ significantly at *P* = 0.05 according to Duncan's range test.

In eggplant fruit, ANOVA revealed that 7 or 14 days exposure to dittany vapours suppressed* B. cinerea* development (up to 94% and 98%, resp.) while increasing DIT concentration resulted in greater fungal inhibition (Figure 3). Interesting, in fruits preexposed to volatiles before fungal inoculation, DIT application induced fruit resistance against the pathogen, by reduced lesion growth (Figure 4). Fruit displayed no visible symptoms of injury or other abnormalities even at the highest DIT oil concentration employed in the study. In previous studies, strawberries (*Fragaria ananassa* Duch.) treated with EO (0.1 mL/L) of tea-tree oil (*Melaleuca alternifolia* L.) reduced 34% the severity of decay during storage at 10°C as compared to the control [29]. Cassia (*Acacia farnesiana* L.) oil at 0.5 mL/L alone or in combination with MgSO_4_ (0.25–3% w/v) reduced the percentage of decayed tomatoes [30]. Plotto et al. [26] reported that EOs (oregano, thyme, lemongrass, and coriander) vapors (50 mg/L) were not successful in stopping disease (*B. cinerea*,* A. arborescens*,* R. stolonifer,* and* Geotrichum candidum*) development in inoculated tomatoes. Additionally, some oil vapors appeared to induce phytotoxicity (introduced in different ratios or might be due to one or more compounds present in the oil) on treated fruit under long periods of exposure. This is maybe due to thinner and higher sensitivity of tomato fruit skin comparing with the eggplant fruit. Furthermore, fruit decay decreased in tomato treated with cinnamon or eucalyptus oil (0.05–0.5 mL/L) vapours and transfer to chilled air [17]. Preexposing tomato fruit to 0.5 mL/L cinnamon vapours for 3 days and then inoculation with fungi reduced* B. cinerea* and* C. coccodes* lesion development, extending “memory” effects [9]. Considering the fungal inhibition observed under vapour treatments, it was suggested that hydroxyl groups in antimicrobial compounds can form hydrogen bonds with active enzymes resulting in deactivation and affecting the biosynthesis of mycotoxins [31].

Many natural substances may play a fundamental role in the host plant/pathogen relationship by enhancing the natural resistance of the fruit to the pathogen. In this sense, fruit induced resistance may have occurred by triggering the increased accumulation and expression of pathogenesis-related proteins (chitinases and glucanases) and/or heat shock proteins [32]. Stimulation of antioxidant properties (ascorbate and phenolic concentration) exhibited by fruit [27] could also be involved.

Interestingly, ANOVA revealed spore production of* B. cinerea* to be significantly (*P* < 0.05) reduced during exposure of wound-inoculated fruits to DIT oils, for all the examined concentrations. Spore production was suppressed following wound inoculation on eggplant fruits preexposed to DIT vapours whereas 250 *μ*L/L treatments revealed the greatest (up to 90%) reduction. DIT vapour treatment (with 100 *μ*L/L or 250 *μ*L/L) of fruits suppressed spore viability by 3% and 100%, respectively, during sustained vapour enrichment. However, spore viability did not differ in pretreated fruits with the vapours (Table 3). The mode of action of volatiles on fungal spores is poorly understood. However, spore morphology, moisture content, and substrate are considered important determinants of fruit susceptibility. Decreased spore germination/production, as highlighted in the present study, would suppress the spread of the fungus and hence its capacity for spore production, making natural products of great importance as a postharvest sanitizers in the storage atmosphere and on surfaces.

### 3.3. Effects of Volatile Vapour Enrichment on Fruit Quality

**Figure 5:**
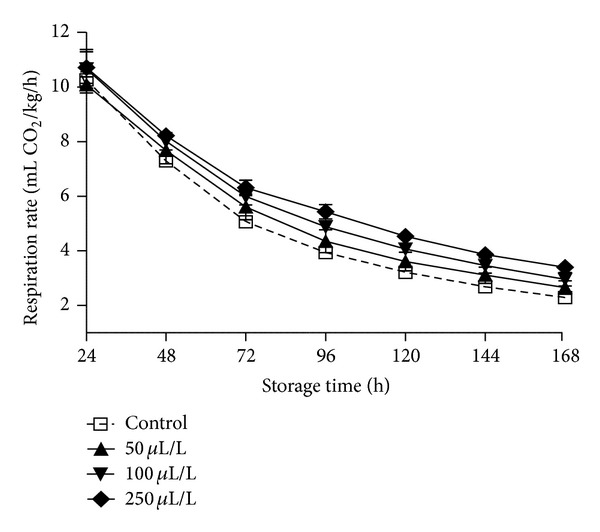
Impacts of dittany essential oil-enrichment (control or volatiles; 50-100-250 *μ*L/L) on respiration rates of eggplant fruits. Fruits were maintained throughout at 12°C and 95% RH. Values represent mean (±SE) of three measurements made on three independent containers per treatment.

Eggplant fruits exposed to DIT vapours had little or no effect on fruit quality (Table 4 and Figure 5). Thus DIT oil in 50 or 100 *μ*L/L increased fruit skin lightness (*L* value averaged 24.68) comparing with the untreated fruits (control treatment, averaged 23.47) with evidence the increased (>86) pulp lightness (more whiteness- Table 4), considered as good quality pulp, being in accordance with previous studies [33], while further evaluation is necessary for the possible changes in the biochemical mechanisms involved. Fruit weight loss did not change after DIT application as marked below 1.07%. No significant differences were observed in fruit firmness while an increased tendency for firmness marked as DIT oil concentration increased, and this possible is related due to surface differentiation comparing with other commodities (i.e., tomatoes; strawberries) that fruit firmness maintained/increased with essential oil application [17]. Additionally, total soluble solids, titratable acidity, and pH of fruit juice as well as calyx colour (*L*, *C*, *h* values) and respiration rates did not differ among treatments (Table 4 and Figure 5). In previous studies, tomato fruits exposed to origanum oil increased weight loss (up to 57%) and soluble sugars for treated fruits comparing with the control, and this is possible related to the different fruits and skin thickness [16]. Moreover, several antioxidative parameters (i.e., ascorbic acid, total phenols, and lycopene) increased following essential oil application in tomato fruits [16, 34], indicating the induced resistance role of essential oils onto the fruits. Fruit firmness on cherries and grapes was affected after exposure to eugenol, thymol, or menthol vapours [11, 35] whereas acetaldehyde vapour-treated avocado delayed fruit softening [13].

This study revealed that volatile-enrichment markedly reduced spoilage by gray mould during the vegetative (mycelium growth) and reproductive phase (spore germination/production) of the fungus, which is of great importance for the disease cycle and spread. Interestingly, the benefits associated with volatiles enrichment were retained in fruits preexposed to vapours, resulting in suppression in lesion growth and spore production. These responses to vapours indicated that vapour treatments may exert a “memory or long-lasting” effect, potentially through the “priming” of fruit tissue responses to subsequent challenge [36] and possible increasing defence-related mechanisms in fruit. Previous studies reported the beneficial effects of lemongrass oil (*Cymbopogon flexuosus *L.) or methyl jasmonate (MJ) on pre- and postinoculation treatment against fruit rotting, onapple (*Malus pumila* L.) and tomato, respectively [17, 37], while studies on tomatoes, strawberries, and cucumbers confirm the antifungal activity of essential oils derived by oregano (*O. vulgare *L. ssp.* hirtum*), thyme (*T. vulgaris *L.), and lemon (*Citrus limon *L.) plants, against some important postharvest pathogens (*B. cinerea*,* Penicillium italicum, *and* Penicillium digitatum*) [38], being in accordance with the present findings. Additionally, several essential oil revealed antifungal effects on apricots, nectarines, tomatoes, and plums [10, 39, 40] demonstrated that the essential oils are potential and promising antifungal agents which could be used as biofungicide in the protection of fresh commodities. The mechanism involved in this response remains, however, unclear.

## 4. Conclusions 

The findings of the current study may have considerable commercial significance, but first efficacy must be proven in microbial studies (both* in vitro* and* in vivo*) optimising volatiles concentrations for specific fresh commodities and in a commercial context where produce is submitted to vapour enrichment in the usual storage/transit bins, cartons, or boxes. To our knowledge, this is the first report about essential oil application for eggplant fruit with considerable benefits for storage conditions as well as putative commercialization in future. Coating fruits with plant volatiles and/or modified atmosphere packaging on a wider range of fresh produce must also be included. Considerable care is needed on application of the essential oils as a continuous vapour treatment and on the low concentration to prevent tainting and undesirable smell of the product. Thin skinned products, not surprisingly, are more prone to tainting than those with thicker skins, so further optimization is necessary for fresh produce common storage. Natural volatiles and essential oils are not as broad spectrum as synthetic pesticides, but their effectiveness is promising and can be improved by using them in conjunction with carefully designed packaging.

## Figures and Tables

**Table 1:** The experimental design of the volatiles impacts of dittany essential oil (DIT; 0–50–100–250 *μ*L/L) on gray mould (*Botrytis cinerea*) development *in vitro* (PDA medium) or *in vivo* (eggplant fruit). In sustained vapour enrichment, fruits (or plates) were inoculated with *B. cinerea* and then exposed to vapours. A second sample of fruits (or plates) was preexposed to vapours, inoculated with fungi, and transferred/stored to “ambient air” (AA; control). Treatments were maintained throughout at 12°C and 95% RH.

	Conditions	Treatment	Section
*In vitro *			
Colony growth (SE)	at 20°C for 2 h, at 12°C for 5 d	Inoc.-5 d in VOL	Section 2.3
Colony growth, transfer (SE)	at 20°C for 2 h, at 12°C for 5 d, at 12°C for 5 d	Inoc.-5 d in VOL-transfer in PDA-5 d in AA	Section 2.3
Colony growth (ME)	at 20°C for 2 h, at 12°C for 5 d, at 12°C for 5 d	5 d in VOL-inoc.-5 d in AA	Section 2.3
Spore production (SE)	at 12°C for 14 d	Inoc.-14 d in VOL	Section 2.6
Spore production (ME)	at 12°C for 5 d, at 12°C for 14 d	5 d in VOL-inoc.-14 d in AA	Section 2.6
Spore germination (SE)	at 12°C for 14 d, at 12°C for 24 h	Inoc.-14 d in VOL-Inoc. in PDA-24 h in AA	Section 2.7
Spore germination (ME)	at 12°C for 5 d, at 12°C for 14 d, at 12°C for 24 h	5 d in VOL-inoc.-14 d in AA-Inoc in PDA-24 h in AA	Section 2.7
*In vivo *			
Lesion growth (SE)	at 20°C for 2 h, at 12°C for 14 d	Inoc.-7 d in VOL	Section 2.4(a)
Lesion growth (ME)	at 20°C for 2 h, at 12°C for 5 d, at 12°C for 7 d	5 d in VOL-inoc.-7 d in AA	Section 2.4(b)
Spore production (SE)	at 12°C for 14 d	Inoc.-14 d in VOL	Section 2.5
Spore production (ME)	at 12°C for 5 d, at 12°C for 14 d	5 d in VOL-inoc.-14 d in AA	Section 2.5
Spore germination (SE)	at 12°C for 14 d, at 12°C for 24 h	Inoc.-14 d in VOL-Inoc. in PDA-24 h in AA	Section 2.6
Spore germination (ME)	at 12°C for 5 d, at 12°C for 14 d, at 12°C for 24 h	5 d in VOL-inoc.-14 d in AA-Inoc in PDA-24 h in AA	Section 2.6

VOL: volatiles; SE: sustained effect; ME: memory effect; PDA: potato dextrose agar; Inoc.: inoculation; AA: ambient air.

Letters a, b under the “section” heading correspond with sections in the text.

**Table 2:** The chemical and percentage composition of the major components from the essential oil of *Origanum dictamnus*.

Number	Compound	R.I.^b^	Retention time	Percentage (%) composition
1	*a*-Thujene	930	9.507	0.68
2	*a*-Pinene^a^	939	9.746	0.56
3	Sabinene	975	11.253	0.06
4	*b*-Pinene^a^	979	11.353	0.10
5	1-Octen-3-ol	979	11.530	0.10
6	*b*-Myrcene	990	11.933	1.12
7	*a*-Phellandrene	1002	12.402	0.10
8	*a*-Terpinene	1017	12.855	1.48
9	p*-*cymene^a^	1024	13.163	12.65
10	Limonene^a^	1029	13.302	0.37
11	*g*-Terpinene^a^	1059	14.400	7.11
12	*cis*-Sabinene hydrate	1070	14.701	0.72
13	Linalool^a^	1096	15.841	0.54
14	Terpinen-4-ol	1177	18.479	0.29
15	Thymoquinone	1252	20.816	0.19
16	Thymol^a^	1290	22.007	0.11
17	Carvacrol^a^	1299	22.382	70.01
18	*a*-Cubenene	1348	23.768	0.08
19	*a*-Copaene	1376	24.565	0.58
20	*b*-Caryophyllene	1419	25.883	1.39
21	**β**-Bisabolene	1505	27.302	0.14
22	**δ**-Cadinene	1523	29.561	0.29
23	Thymohydroquinone	1555	30.761	1.00
24	Caryophyllene oxide	1583	32.548	0.20
	Total (%)			**99.87**

	*Monoterpene hydrocarbons *			24.33
	*Oxygenated monoterpenes *			72.86
	*Sesquiterpene hydrocarbons *			2.68

^a^Identification by comparison of retention times and co-injection with authentic compound.

^b^R.I. (Retention Indices) from experimental using a SBP-5 column using a homologous series of n-alkanes (C9–C25).

**Table 3:** Effects of dittany (DIT) essential oil enrichment (Control or volatiles; 50–100–250 *μ*L/L) on grey mould (*Botrytis cinerea*) spore production and spore germination *in vitro* (PDA medium) or *in vivo* (eggplant fruit). In sustained vapour enrichment, fruits (or plates) were inoculated with *B. cinerea* and then exposed to vapours. In vapour-induced “memory” effect, fruits (or plates) were preexposed to vapours, inoculated with fungi, and transferred/stored to “ambient air.” Treatments were maintained throughout at 12°C and 95% RH. In each column, mean values (*n* = 12) of fruits (*n* = 6) of plates for the individual vapour enrichment followed by the same letter do not differ significantly at *P* = 0.05 according to Duncan's range test.

DIT	Sustained vapour enrichment		Vapour-induced “memory” effect	
	Spore production (10^5^/mL)	Spore germination (%)	Spore production (10^5^/mL)	Spore germination (%)
*In vitro *				
0	22.70^a^	89.33^a^	33.25^a^	98.66^a^
50	21.57^a^	40.66^b^	16.31^b^	97.83^a^
100	0.00^b^	—^Z^	0.00^c^	—
250	0.00^b^	—	0.00^c^	—
*In vivo *				
0	69.94^a^	99.00^a^	51.67^a^	99.08^a^
50	1.46^b^	98.16^a^	16.67^b^	97.41^a^
100	0.06^b^	96.58^b^	10.77^c^	97.00^a^
250	0.00^b^	—	5.49^d^	97.41^a^

^Z^—implicated that spore germination could not be measured as spores have been not produced.

**(a):** 

DIT	Weight loss (%)	Firmness (kg cm^−2^)	TSS (°Brix)	TA (% citric acid)	pH
0	1.06^a^	13.52^a^	4.01^a^	0.77^a^	5.63^a^
50	1.07^a^	13.34^a^	3.88^a^	0.76^a^	5.50^a^
100	0.97^ab^	14.61^a^	3.95^a^	0.77^a^	5.54^a^
250	0.88^b^	15.59^a^	3.70^a^	0.75^a^	5.56^a^

**(b):** 

DIT	Colour						
	Calyx			Skin			Pulp
	*L*	*C*	*h*	*L*	*C*	*h*	*L*
0	54.88^a^	29.87^a^	113.08^a^	23.47^b^	3.73^a^	355.68^bc^	82.74^c^
50	54.72^a^	30.03^a^	114.56^a^	24.22^a^	4.63^a^	357.27^a^	84.44^bc^
100	54.52^a^	29.72^a^	114.25^a^	24.68^a^	4.21^a^	355.39^c^	85.10^ab^
250	54.07^a^	31.21^a^	114.60^a^	24.08^ab^	4.48^a^	357.00^ab^	86.60^a^
